# Effects of Fishmeal Replacement by *Clostridium Autoethanogenum* Protein Meal on Cholesterol Bile Acid Metabolism, Antioxidant Capacity, Hepatic and Intestinal Health of Pearl Gentian Grouper (*Epinephelus Fuscoguttatus* ♀ × *Epinephelus Lanceolatus* ♂)

**DOI:** 10.3390/ani13061090

**Published:** 2023-03-18

**Authors:** Bocheng Huang, Menglin Shi, Aobo Pang, Beiping Tan, Shiwei Xie

**Affiliations:** 1 Laboratory of Aquatic Animal Nutrition and Feed, College of Fisheries, Guangdong Ocean University, Zhanjiang 524088, China; 2 Aquatic Animals Precision Nutrition and High-Efficiency Feed Engineering Research Centre of Guangdong Province, Zhanjiang 524088, China; 3 Key Laboratory of Aquatic, Livestock and Poultry Feed Science and Technology in South China, Ministry of Agriculture, Zhanjiang 524088, China; 4 Guangdong Provincial Key Laboratory of Aquatic Animal Disease Control and Healthy Culture, Zhanjiang 524088, China

**Keywords:** *Clostridium autoethanogenum*, fishmeal replacement, pearl gentian grouper, cholesterol bile acid metabolism, antioxidant capacity, hepatic health, intestinal health

## Abstract

**Simple Summary:**

With the development of the breeding industry, the production of fishmeal will not be able to meet the needs of feed production in the future. *Clostridium autoethanogenum* protein meal (CAP) is a by-product of people using carbon monoxide exhaust from the steel industry to produce ethanol, with a potential to become a substitute ingredient for fishmeal to alleviate the shortage of fishmeal. This study was conducted on pearl gentian grouper (*Epinephelus fuscoguttatus* ♀ × *Epinephelus lanceolatus* ♂), an economic fish species widely cultured in Southeast Asia. After using different concentrations of CAP to replace fishmeal in the feed for eight weeks, respectively, we found that it could replace 45% of fishmeal in the pearl gentian grouper feed through growth performance, various physiological and biochemical indexes analysis experiments, but a 60% replacement level would significantly affect health and growth. These findings provide a reference for the promotion and further research of CAP as a new environmentally friendly ingredient, which is positive for the sustainable development of the farming industry and even for the utilization of waste gas from the steel industry.

**Abstract:**

In this study, we present data from an eight-week growth trial with pearl gentian grouper fed either a reference diet (FM) with a fishmeal level of 50%, or test diet wherein 15% (CAP15), 30% (CAP30), 45% (CAP45), and 60% (CAP60) fishmeal was replaced by *Clostridium autoethanogenum* protein meal (CAP). Results showed that the weight gain and daily feed intake ratio of CAP60 were significantly lower than the FM group. In the serum, compared to the FM group, the content of malondialdehyde (MDA), the activities of alanine aminotransferase in CAP60 and CAP45 groups, and acid phosphatase in the CAP60 group were significantly higher, while the content of total cholesterol in CAP60 and CAP45 groups was significantly lower. In the liver, compared to the control group, the content of MDA in the CAP60 group was significantly higher. *3-hydroxy-3-methylglutaryl coenzyme A reductase* in CAP30 to CAP60 groups and *farnesoid X receptor* in CAP60 were significantly upregulated. In distal intestines, the activities of trypsin and superoxide dismutase of CAP30 to CAP60 groups were significantly lower than the FM group. In conclusion, for pearl gentian grouper, CAP could replace up to 45% of the fishmeal in the feed, while a 60% replacement level will affect cholesterol bile acid metabolism and health.

## 1. Introduction

Pearl gentian grouper (*Epinephelus fuscoguttatus* ♀ × *Epinephelus lanceolatus* ♂), with the heterosis of fast growth and strong disease resistance, is farmed in large quantities in China [1]. It is a predatory fish which requires about 45% fishmeal in the diet [2]. Fishmeal is an expensive ingredient with limited supply. There have been many studies to assess the feasibility of replacing fishmeal with other ingredients [3].

Single-cell protein generally refer to the biological proteins processed by microalgae, fungi, and bacteria, which high contents of crude protein and are rich in amino acids, various vitamins, trace elements, and bioactive substances [4]. Single-cell protein can be produced from agricultural by-products, oil industry waste gas [5], or through further processing of fermentation residues from the brewing and monosodium glutamate manufacturing industries [6]. The full utilization of single-cell protein can lead to resource conservation, and in recent years, it has been widely studied as a promising alternative to fishmeal substitute in aquafeed [7].

*Clostridium autoethanogenum* is a strictly anaerobic, gram-positive, rod-like, motile bacterium first reported in 1994 by Abrini et al. [8]. It is capable of producing ethanol using CO as a carbon source and is therefore used to treat exhaust gases from the steel industry and to produce ethanol [9]. The mash produced during this fermentation process is extracted and then processed to produce a protein ingredient, called *Clostridium autoethanogenum* protein (CAP). CAP crude protein content is higher than 80%, amino acid composition is similar to fishmeal, and no anti-nutritional factors have been detected [5]. Moreover, the results of genomic sequences showed that *Clostridium autoethanogenum* had no virulence genes [10], so CAP is considered a viable alternative to fishmeal. It is worth noting that CAP lacks crude fat compared to fishmeal [11] and also lacks phosphorus when used in shrimp feed [12], which needs to be compensated by additional supplementation. In previous studies, the proportion of CAP substituted fishmeal can reach 63% [13], 50% [14,15], or 42.8% [16] in *Micropterus salmoides*, 58.0% in *Acanthopagrus schlegelii* [5], 30% in *Larimichthys crocea* [17], and 30% in *Litopenaeus vannamei* [18], while higher levels of substitution would significantly reduce the growth and had varying impacts on the liver or intestinal health of aquatic animals. In one of the studies on *M. salmoides*, it was shown that 75% CAP substitution level causes swelling of hepatocytes [13], while according to another study, more than 57.14% CAP substitution levels caused morphological atrophy of the intestine in *M. salmoides.* CAP substitution levels above 60% also cause upregulation of *tumor necrosis factor α* (*tnf-α*) and downregulation of *interleukin 10* (*il-10*) in the intestine of *L. crocea* [17].

Up to now, the application of CAP as a kind of ingredient for grouper has not been reported. This study aimed to investigate the feasibility and suitable substitution amount of fishmeal in the feed of pearl gentian grouper by replacing it with CAP. In addition to growth performance, physiological and biochemical indices, gene expression, and morphology of the tissues of grouper were monitored and evaluated comprehensively in this study.

## 2. Materials and Methods

### 2.1. Diets and Experimental Design

The control group feed was made with gluten (wheat, crude protein: 73.75%; crude fat: 0.11%), soybean meal (crude protein: 68.21%; crude fat: 9.00%), brown fishmeal (crude protein: 68.21%, crude fat: 9.00%) as protein ingredients and wheat flour (crude protein: 9.19%, crude fat: 0.38%) as the main binder, which was named FM. Four experimental diets were formulated to be isonitrogenous and isolipidic with 15%, 30%, 45%, and 60% fishmeal replaced by CAP (crude protein: 84.14%, crude fat: 0.19%, produced by Shoulang Biotechnology Co., Ltd. Beijing, China), named CAP15, CAP30, CAP45 and CAP60, respectively. Methionine and arginine were added in the experimental diets to the same level as the control group. The nutritional composition of CAP and fishmeal is shown in supplementary material Table S1.

Except for choline chloride, all ingredients were thoroughly mixed after squeezing through a 60-mesh sieve and then mechanically mixed with choline chloride and water. The 2.5 mm diameter pellets were extruded using a twin-screw extruder (F-26, South China University of Technology, Guangzhou, China) then air-dried for 48 h, and finally, stored at −20 °C before use. The formulation and proximate composition of the experimental diets are shown in Table 1.

### 2.2. Experimental Animal and Feeding Management

The experiment was carried out in the Zhanjiang Hi-Tech Park of Ocean (Zhanjiang, China). The juvenile pearl gentian grouper for the experiment were obtained from a local breeding factory (Zhanjiang, China). A total of 500 fish (18.01 ± 0.82 g) were randomly distributed into 20 tanks (0.3 m^3^) at a density of 25 fish per tank, and each diet was assigned to four tanks. Fish were fed to satiation at 8:00 and 17:00 each day. Continuous aeration was provided to each tank. The water was changed once a day, the temperature was 26 to 30 °C, and the salinity was 24 to 32%. Dead fish were weighed and the mortalities were recorded. The feeding trial lasted for 8 weeks.

### 2.3. Sample Collection

Fish from each tank were starved for 24 h, then counted and weighed. Three fish per tank were randomly selected to record body weight, visceral weight, and hepatic weight to calculate the VSI and HSI. Six fish per tank were randomly selected for blood sampling from the caudal vein. The blood samples were kept in the ice box. The front positions of liver and part of the distal intestines taken from two fish per tank were cut off and stored in 4% formaldehyde solution for hematoxylin-eosin (H&E). For FM, CAP30, and CAP60 groups, the other part of the distal intestines was cut to 1 mm in size, and stored in 2.5% glutaraldehyde solution for transmission electron microscopy (TEM) samples preparation. The liver, dorsal muscle, and distal intestine samples from four fish per tank were collected, immediately frozen in liquid nitrogen, and then stored at −80 °C for analysis of the biochemical indices, enzyme activities, and gene expression. Three fish were taken from each tank and used for proximate composition assay.

### 2.4. Proximate Composition Assay

Proximate composition of experimental diets and whole fish samples were measured. Moisture was analyzed by drying the samples to constant weight at 105 °C. Crude lipid was measured by petroleum ether using the Soxhlet method. Crude protein was determined using the Dumas Nitrogen method by Primacs 100 analyzer (Skalar, Dutch) and estimated by multiplying nitrogen by 6.25.

### 2.5. Biochemical Indices and Enzyme Activities Assay

After 12 h of sampling, the blood samples were centrifuged (3500 rpm, 15 min, 4 °C), then serum was obtained. About 200 mg of tissue was weighed and then homogenized in 9:1 (saline solution: tissue) saline solution with ice bath. After centrifugation (2500 rpm, 10 min, 4 °C), supernatants were collected.

For serum samples, the contents of total protein (TP), triglyceride (TG), uric acid (UA), total cholesterol (T-CHO), malondialdehyde (MDA), glutathione (GSH), the activities of acid phosphatase (ACP), alanine aminotransferase (ALT), aspartate aminotransferase (AST), as well as total antioxidant capacity (T-AOC) were measured. For the treated liver samples, the contents of MDA, GSH, the activities of ACP, superoxide dismutase (SOD) and lysozyme, as well as total antioxidant capacity (T-AOC) were measured. For the treated distal intestine samples, the activities of trypsin, SOD, and lysozyme were measured.

Among the above indices, except for the trypsin, SOD and lysozyme were determined by enzyme-linked immunosorbent assay (ELISA) detection kits (Shanghai Enzyme-linked Biotechnology, Shanghai, China), following the instructions. All the other indices were determined by detection kits (Nanjing Jiancheng Bioengineering Institute, Nanjing, China), following the instructions.

### 2.6. Tissue Morphological Histology

According to the method of Chen et al. [19], the samples for H&E stain were fixed with paraffin and sectioned, dewaxed, and stained with Hematoxylin solution, then were dehydrated and sealed with neutral gum. The histology of samples was photographed using an optical microscope (Nikon Ni-U, Japan). For the hepatic sections, nuclear excursion, inflammatory, and ballooning degenerated hepatocytes were assessed. For the distal intestines sections, ten mucosal folds were randomly selected, the height and width were measured, and the number of goblet cells within 200 µm from the top was counted. Ten measurement points were selected at equal intervals to measure the thickness of the muscularis.

The electron microscopy samples were prepared and observed with reference to the method of Huang et al. [20]. Briefly, tissue samples were fixed in 2% osmium tetroxide phosphate buffer, then dehydrated in ethanol, an embedded in epoxy resin before being cut into ultrathin sections by an ultramicrotome (Leica EM UC7, Japan). Ultrathin sections were dyed by uranyl acetate and lead citrate, and photographed using a transmission electron microscope (HITACHI HT7600, Japan) at an accelerating voltage of 80 kV. Thirty intestinal microvillus were randomly selected in each sample to measure the length, and the number of microvillus within 10 μm was counted to calculate the density.

### 2.7. Real-Time Quantitative PCR Assay

Total RNA was extracted from tissue using an RNA extraction kit (Transgen Biotech, Beijing, China), and the concentration was detected with a NanoDrop 2000 (Thermo Fisher Scientific, Waltham, MA, USA) and adjusted to 600 ng μL^−1^ by adding ultrapure water. Evo M-MLV RT kit (Accurate Biology, Changsha, China) was used to synthesize cDNA. The methods and reactions followed the instructions.

The amplification of real-time quantitative PCR was performed in a total volume of 10 μL, comprising 0.1 μL (5μM) of each primer, 1 μL of cDNA, 3.8 μL of sterilized double-distilled water, and 5 μL of 2X SYBR^®^ Green Pro Taq HS PremixII (Accurate Biotechnology, China), using a quantitative thermal cycler (Light Cycler 480, Roche Diagnostics, Switzerland). The target gene expression was calculated by the 2^−∆∆Ct^ method [21] using *β*-*actin* as the reference gene [22] and the FM group as the reference group. The information of primers was presented in Table 2. Some of these primers were derived from the studies of Xu et al. [23] and Yin et al. [24], which were designed according to the full-length sequences from the sequenced transcriptome of the pearl gentian grouper (not published).

For the aspect of cholesterol bile acid metabolism, four genes, *3-hydroxy-3-methylglutaryl coenzyme A reductase* (*hmgcr*), *farnesoid X* (*fxr*) and *cholesterol 7α-hydroxylase* (*cyp7a1*), *cholesterol 27α-hydroxylase* (*cyp27a1*) were selected. For the IGF-1/PI3K/AKT/mTOR signaling pathway, six genes, *insulin-like growth factor* (*igf-1*), *serine/threonine-protein kinase* (*akt*), *mammalian target of rapamycin* (*mtor*), *eukaryotic translation initiation factor 4E-binding protein 1* (*4e-bp1*), and *ribosomal protein S6 kinase 1* (*s6k1*) were selected. For the Nrf2-Keap1 signaling pathway, *nuclear erythroid 2-related factor 2* (*nrf2*) and *kelch-like ECH associating protein 1* (*keap1*) were selected.

### 2.8. Calculations and statistical analysis

The calculation formulas were as follows:

1. Weight gain (WG,%) = 100 × (W_t_ − W_i_)/W_i_;
2. Specific growth rate (SGR,%) = 100 × (lnW_t_ − lnW_i_)/t;
3. Survival rate (SR,%) = 100 × N_t_/N_i_;
4. Feed conversion ratio (FCR) = W_fi/_(W_t_ − W_i_)
5. Daily Feed intake ratio (DFI,%/day) = 100 × W_fi_/((W_g_ − W_i_)/2 × t)
6. Condition factor (CF, kg/cm^3^ %) = 100 × Body weight (g)/Body length (cm^3^)
7. Hepatosomatic index (HSI,%) = 100 × Hepatosomatic weight (g)/Body weight (g)
8. Viscerasomatic index (VSI,%) = 100 × Viscerasomatic weight (g)/Body weight (g)

where W_t_ is final body weight (g); W_i_, initial body weight (g); W_fi,_ Total feed intake (g); N_t_, the final fish number; N_i_, the initial fish number; t, the duration of experiment days.

All data were subjected to statistical verification using one-way analysis of variance (ANOVA) with SPSS 25.0 (International Business Machines Corp, Armonk, NY, USA). When there were significant differences between groups, Tukey’s multiple comparison test was performed with differences significant at *p* < 0.05. Further, a follow-up trend analysis was performed using orthogonal polynomial comparison, to determine whether the effects were linear or quadratic [17].

## 3. Results

### 3.1. Growth Performance

Growth performance is presented in Table 3. CAP substitution of 15–45% fishmeal in the diet had no significant effects on FBW, WG, and SGR of grouper, but for fish fed a CAP60 diet, all these indicators were significantly lower than fish fed the control and CAP15 diets (*p* < 0.05). With the increase of dietary CAP levels, the FBW, WG, SGR, and DFI showed both significantly negative linear and quadratic patterns (*p* < 0.05). In addition, the CAP30 group had the highest DFI, which was significantly higher than the CAP60 group (*p* < 0.05). There were no significant differences in the FCR and SR between each group.

### 3.2. Morphometric Parameters and Whole Fish Composition

As shown in Table 4, in morphometric parameters, with the increase of dietary CAP levels, HSI and VSI showed significantly positive linear and quadratic patterns (*p* < 0.05). HSI of fish fed CAP60 diet was significantly higher than other groups; VSI of fish fed CAP60 diet was significantly higher than the control group (*p* < 0.05). For body compositions, moisture, crude protein, and crude lipid of whole fish did not show any significant difference among each group (*p* > 0.05).

### 3.3. Serum Biochemical Indices and Enzyme Activities

The results of serum biochemical indices and enzyme activities are presented in Table 5. With the increase of dietary CAP levels, the contents of T-CHO and GSH showed both significantly negative linear and quadratic patterns (*p* < 0.05). Among them, the content of T-CHO of fish fed CAP15, CAP45, CAP60 diets were significantly lower than the in the control group, and in addition, the content of T-CHO of fish fed CAP45, CAP60 diets also had significantly lower than in the CAP30 group (*p* < 0.05). The content of GSH of fish fed CAP60 diet were significantly lower than the CAP15 group (*p* < 0.05). The content of MDA and the activity of ALT of fish fed CAP30, CAP45, CAP60 diets, the activity of ACP of fish fed CAP60 diet were significantly higher than the control group, also the content of MDA of fish fed CAP45 and CAP60 diets were significantly higher than the CAP15 group, showed both significantly positive linear and quadratic pattern (*p* < 0.05). As the level of dietary CAP increased, the activity of AST showed a significantly positive quadratic pattern, with the fish fed CAP45 were significantly higher than those fed the CAP60 diet (*p* < 0.05). There were no significant differences in the contents of TP, TG, UA, and T-AOC among each group (*p* > 0.05).

### 3.4. Biochemical Indices and Enzyme Activities in the Liver and Distal Intestines

Table 6 shows the biochemical indices and enzyme activities in the liver and distal intestines. In liver, the content of MDA of fish fed a CAP60 diet and the activity of ACP of fish fed a CAP45 diet were significantly higher than the FM and CAP15 groups, showed both significantly positive linear and quadratic patterns (*p* < 0.05). The content of GSH of fish fed CAP60 diet was significantly lower than the FM and CAP15 groups; theT-AOC of fish fed CAP60 diet was significantly lower than the control group, and showed both significantly negative linear and quadratic patterns (*p* < 0.05). In addition, the activity of SOD and lysozyme was not significantly different between each group (*p* > 0.05).

In distal intestines, with the increase of dietary CAP levels, the activities of trypsin and SOD of fish were significantly lower than the control group when 30% or more fishmeal was replaced in the diet, and showed both significantly negative linear and quadratic patterns (*p* < 0.05). However, there were no significant differences in the activity of lysozyme among each group (*p* > 0.05).

### 3.5. Hepatic and Distal Intestinal Morphological Observation

**Figure 1:**
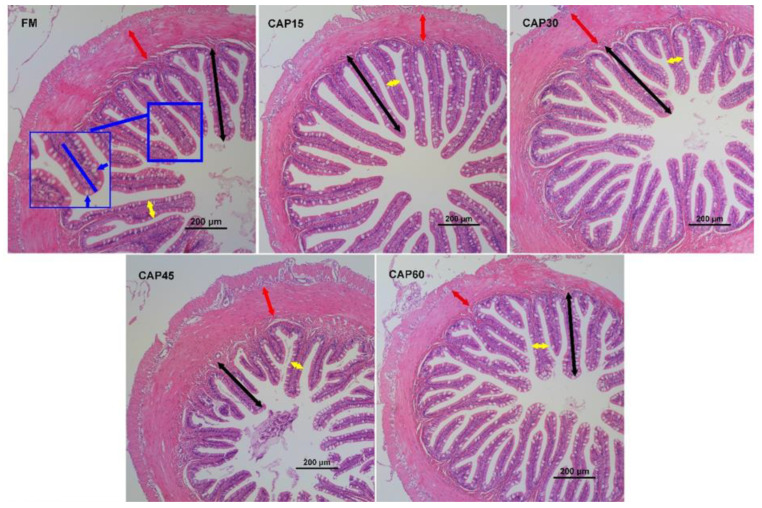
HE-stained sections of distal intestines of pearl gentian grouper fed gradient substitution of fishmeal in the diet with CAP (magnification = 40, Scale size = 50 μm).

Figure 1 shows the images of HE-stained sections of distal intestines, and Table 7 shows the morphological histology indices. With the increase of dietary CAP, the height of mucosal fold, the thickness of muscularis, and the relative number of goblet cells showed both significantly negative linear and quadratic patterns (*p* < 0.05). Among them, the height of mucosal fold and the relative number of goblet cells of fish fed CAP60 diet were significantly lower than the FM and CAP15 groups, the thickness of muscularis of fish fed CAP60 diet were significantly lower than the fish fed a CAP15 diet (*p* < 0.05).

**Figure 2:**
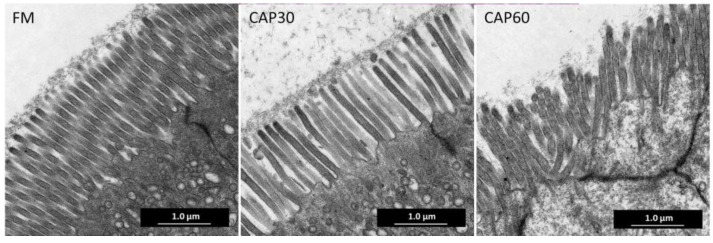
TEM of distal intestines of pearl gentian grouper-fed gradient substitution of fishmeal in the diet with CAP (magnification = 6000, Scale size = 1 μm).

Figure 2 shows the TEM of distal intestines, and Table 8 shows the morphological histology indices. The microvillus height of fish fed the CAP60 diet was significantly lower than those in the control group, and the microvillus density of fish fed the CAP60 diet was significantly lower than the FM and CAP30 groups, showing a significant positive linear and quadratic trend with increasing dietary CAP levels (*p* < 0.05), and appearing disorganized and sparse.

**Figure 3:**
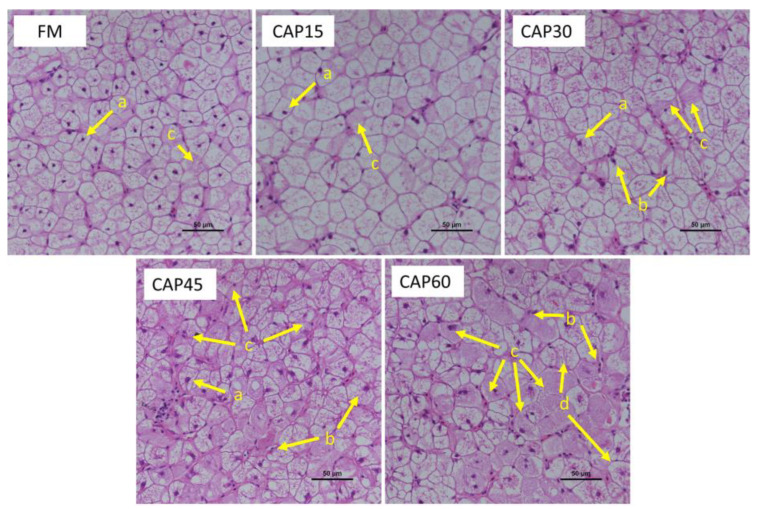
Histological appearance of liver stained with HE of pearl gentian grouper-fed gradient substitution of fishmeal in the diet with CAP (magnification = 400, Scale size = 50 μm). a: normal hepatocyte; b: hepatocyte with nuclear excursion; c: ground-glass hepatocyte; d: ballooning degeneration.

HE-stained sections of the liver under 400 times magnification are shown in Figure 3. Hepatocytes were polyhedral in shape, the nucleus was positioned in the middle, and no obvious inflammatory cell infiltration was observed in fish fed the control and CAP15 diets. Comparatively, more hepatocytes with nuclear excursion were observed in last three groups, and more ground-glass hepatocytes were observed in fish fed the CAP45 diet, and in the CAP60 group, hepatocytes with ballooning degeneration were observed.

### 3.6. Real-Time Quantitative PCR Assay

**Figure 4:**
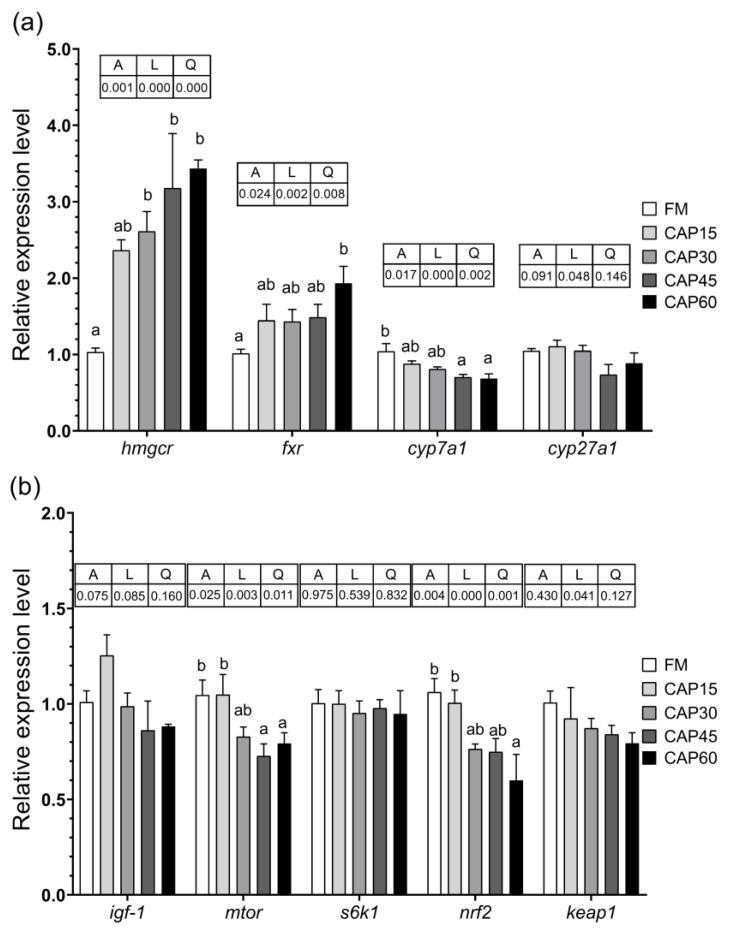
Relative mRNA expression level in liver of pearl gentian grouper fed gradient substitution of fishmeal in the diet with CAP. (**a**). Cholesterol and bile acid metabolization related genes; (**b**). mTOR signaling pathway and Nrf2 and signaling pathway related genes. *hmgcr, 3-hydroxy-3-methylglutaryl coenzyme A reductase*; *fxr*, *farnesoid X receptor*; *cyp7a1*, *cholesterol 7α-hydroxylase*; *cyp27a1*, *cholesterol 27α-hydroxylase*; *igf-1*, *insulin-like growth factor*; *akt*, *serine/threonine-protein kinase*; *mtor*, *mammalian target of rapamycin*; *s6k1*, *ribosomal protein S6 kinase 1*; *nrf2*, *nuclear erythroid 2-related factor 2*; *keap1*, *kelch-like ECH associating protein 1*. Bars represent means ± pooled standard error of the mean (SEM), *n* = 4. Different superscript letters are significantly different (*p* < 0.05) among treatments. Significance probability associated with the F-statistic. A: Analysis of Variance; L: analysis of Linear trend; Q: Analysis of Quadratic trend.

The relative levels of gene expression in the liver are presented in Figure 4. *hmgcr*, *fxr* showed both significantly positive linear and quadratic patterns with the increase of dietary CAP levels, while *cyp7a1*, *mtor*, and *nrf2* showed both a significantly negative linear and quadratic pattern (*p* < 0.05). Compared to the control group, *hmgcr* in CAP30, CAP45, CAP60 groups and *fxr* in the CAP60 group were significantly upregulated, while *cyp7a1* in CAP45and CAP60 groups were significantly downregulated. *mtor* in CAP45, CAP60 groups and *nrf2* in the CAP60 group were significantly downregulated, compared to the FM and CAP15 groups. As the dietary CAP levels increased, *cyp27a1* and *keap1* showed a significantly negative linear pattern (*p* < 0.05). There were no significant differences in the relative expression levels of *s6k1* among each group (*p* > 0.05).

**Figure 5:**
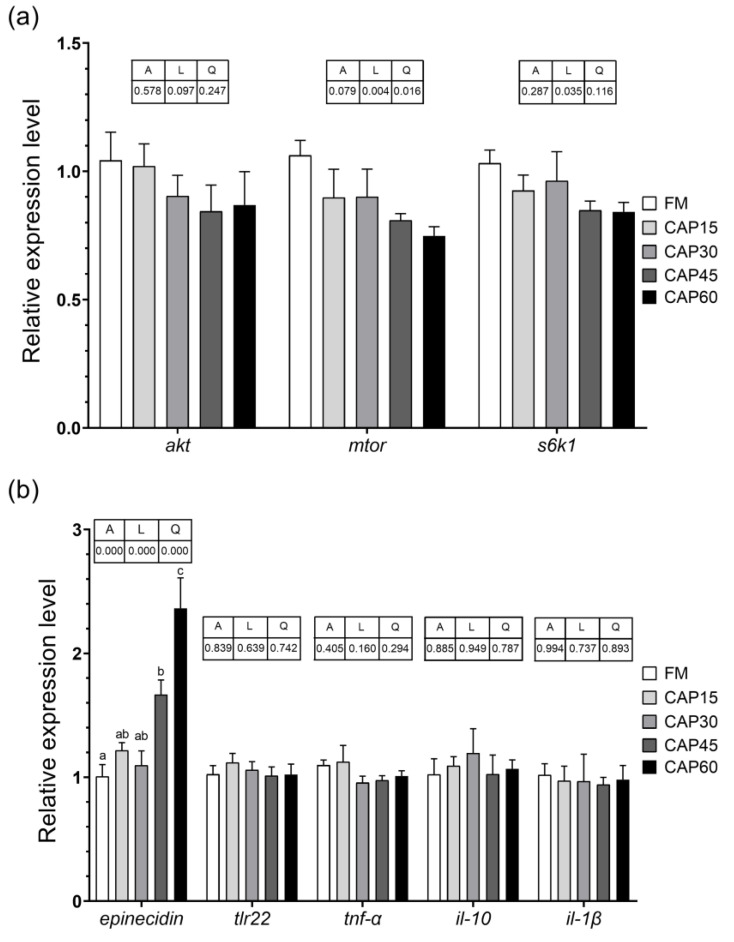
Relative mRNA expression level in distal intestines of pearl gentian grouper fed gradient substitution of fishmeal in the diet with CAP (**a**): mTOR signaling pathway-related genes; (**b**) Epinecidin and inflammatory factor-related genes. *akt*, *serine/threonine-protein kinase*; *mtor*, *mammalian target of rapamycin*; *s6k1*, *ribosomal protein S6 kinase 1*; *tlr22*, *toll-like receptor*; *tnf-α*, *tumor necrosis factor α*; *il-10*, *interleukin 10*; *il-1β*: *interleukin 1β*. Bars represent means ± pooled standard error of the mean (SEM), *n* = 4. Different superscript letters are significantly different (*p* < 0.05) among treatments. Significance probability associated with the F-statistic. A: Analysis of Variance; L: analysis of Linear trend; Q: Analysis of Quadratic trend.

Figure 5 showed the results of gene expression in the distal intestines. With the increase of dietary CAP levels, the relative expression level of *epinecidin* showed both significantly positive linear and quadratic patterns, while *mtor* showed both significantly negative linear and quadratic patterns, while *s6k1* showed only significantly negative quadratic patterns (*p* < 0.05). The relative expression of *epinecidin* in the CAP45 group was significantly upregulated relative to the control group, and further, it also exhibited significant upregulation in the CAP60 group relative to the CAP45 group (*p* < 0.05). Other than that, there were no significant differences in the relative expression levels of *akt*, *toll-like receptor* (*tlr22*), *tnf-α*, *il-10*, and *interleukin 1β* (*il-1β*) among each group (*p* > 0.05).

**Figure 6:**
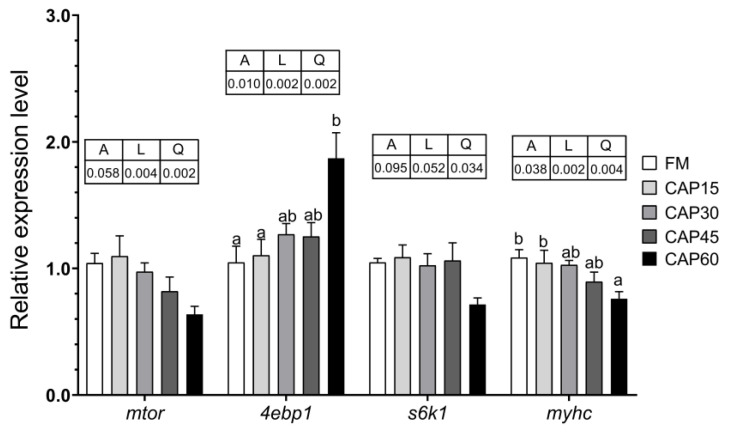
Relative mRNA expression level of protein synthesis-related genes in dorsal muscle of pearl gentian grouper fed gradient substitution of fishmeal in the diet with CAP. *mtor*, *mammalian target of rapamycin*; *4e-bp1*, *eukaryotic translation initiation factor 4E-binding protein 1*; *s6k1*, *ribosomal protein S6 kinase 1*; *myhc*, *myosin heavy chain*. Bars represent means ± pooled standard error of the mean (SEM), *n* = 4. Different superscript letters are significantly different (*p* < 0.05) among treatments. Significance probability associated with the F-statistic. A: Analysis of Variance; L: analysis of Linear trend; Q: Analysis of Quadratic trend.

As shown in Figure 6, with the levels of dietary CAP increased, a negative linear and a quadratic trend was found including the mRNA expression level of *mtor* and *myosin heavy chain* (*myhc*), while *s6k1* showed only a negative linear trend, and *4ebp1* showed a positive linearly and quadratic trend in the dorsal muscle, relatively (*p* < 0.05). Furthermore, compared to the control group and fish fed CAP15 diet the mRNA expression level of *4ebp1* in CAP60 was significantly upregulated, while *myhc* was significantly downregulated (*p* < 0.05).

## 4. Discussion

The results of this study indicate that CAP is a feasible alternative to fishmeal in the feed of pearl gentian grouper. Even a 45% replacement level does not significantly affect the growth of grouper. Similar results have been shown in other studies of marine fish. In a study on *A. schlegelii*, Chen et al. [5] found that CAP could replace 58% of fishmeal without significantly affecting growth indices, while in a study on *L. crocea*, Wu et al. [17] found that CAP could replace 30% of fishmeal in the feed. The studies on *A. schlegelii* have found that replacing fishmeal in feed with high levels of CAP reduced the palatability of the feed [5]. In this trial, the mean value of DFI in the CAP30 group was even higher than that in the control group, which may indicate that feeds with appropriate CAP substitution levels have better feeding attraction. However, when 45% or more fishmeal was replaced in the diet, the DFI of grouper was reduced compared with the control group, but the difference was not significant, indicating that the palatability of CAP is still relatively suitable for grouper. Therefore, the significant reduction of growth performance in the CAP60 group may be influenced by other factors.

ACP is a hydrolase that destroys the structure of pathogens by hydrolyzing phosphate esters to enhance immunity [25]. In this experiment, the activity of ACP in serum and liver increased with the increasing dietary CAP levels, which was contrary to the findings of Wu et al. on *L. crocea* [17], probably due to the difference of species. Another study indicated that ACP activity within the serum and liver of hybrid grouper was increased by negative stress [26], suggesting that the effects of excessive levels in the diet of CAP on grouper may negatively affect the immunity of grouper.

The serum activities of AST and ALT are low in normal conditions, but when liver damage occurs, these two transaminases escape from the hepatocytes, causing a high level of their activities in the serum [27]. GSH is an important antioxidant substance in cells and is essential for maintaining the stability of the intracellular environment [28]. Therefore, when lipid peroxidation increases, the GSH content decreases, in addition to an increase in MDA content [29]. In this experiment, the change of activity of two transaminases, the contents of MDA, GSH in the serum and the changes of MDA, GSH, T-AOC in the liver illustrated the oxidative damage to the organism by excessive substitution of fishmeal by CAP. In a study on *M. salmoides*, Yang et al. [16] also found that fishmeal in CAP replacement feed significantly increased serum MDA levels, similar to this experiment. However, in the study of *A. schlegelii*, 58% CAP substitution levels in diet did not significantly affect the antioxidant capacity of the liver [5]. It is possible that these results are caused by differences in the experimental species.

The Nrf2-Keap1 signaling pathway is an important endogenous signaling pathway affecting the resistance to oxidative stress [30]. Among them, *nrf2* is a transcription factor that can upregulate the transcription of antioxidant-related genes to maintain cellular defense [31]. When the intracellular oxidative damage situation normalizes, *keap1* expression is upregulated and Keap1 enters the nucleus to bind with Nrf2 and exit the nucleus, returning to its steady-state [32]. In studies on *Sillago sihama* [33] and *Pseudosciaena crocea* [34], *keap1* expression level in the liver was positively correlated with *nrf2*. Similar results were presented in this study, and the changes in the expression of these two genes also suggested that the level of CAP substitution to fishmeal in the feed affected the antioxidant capacity of the grouper liver.

In addition to changes in biochemical indicators, the morphological structure of liver cells can be significantly influenced by diet [35]. In histopathology, the position of nuclei can reflect the health of the hepatocytes [36]. The ground-glass microstructure of the hepatocytes indicates hypertrophy of the smooth endoplasmic reticulum, a sign of inflammation [37], while ballooning degeneration of the hepatocytes is often considered a form of apoptosis [38]. This result revealed that substitution of fishmeal in feed with high level CAP induced increased hepatocytes inflammation.

Cholesterol is an important component of cell membranes and a precursor of bile acids and steroid hormone. Since fish can synthesize cholesterol, it is usually considered a non-essential nutrient [39]. Liver is the principal site of cholesterol synthesis, as well as the place of producing bile acids by cholesterol, where HMGCR and CYP7A1, CP27A1 are the key enzymes involved in cholesterol synthesis and bile acid production, respectively [40]. The farnesol X receptor (FXR) is the bile acid receptor that inhibits the expression of *cyp7a1* to negatively regulate the production of bile acids [23]. Although we designed the feed formulation with fish oil to equalize the crude fat in each group, the results showed that the CAP high level replacement group showed differences in cholesterol metabolism compared to the FM group. According to the study by Wu et al. [41], the addition of cholesterol in the diet of giant grouper (*Epinephelus lanceolatus*) resulted in higher blood cholesterol content than the control group, but the expression of *hmgcr* was downregulated as a result. Similarly, in this study with increasing dietary CAP replacement level, the expression level of *hmgcr* was significantly upregulated, while the serum T-CHO content in CAP45 and CAP60 groups was significantly lower than the FM group. Furthermore, the change in the relative expression of *fxr*, *cyp7a1*, and *cyp27a1* indicates that bile acid synthesis is inhibited when high level of fishmeal was replaced by CAP, which may be due to the deficiency of cholesterol. However, more in-depth verification is needed to further investigated.

The mTOR is the signaling pathway involved in regulating the metabolism of nutrients such as proteins, lipids, and nucleic acids in the organism to preserve normal cell growth and proliferation [24]. In the IGF-1/PI3K/AKT/mTOR signaling pathway, IGF-1 activates the PI3K/AKT pathway, which in turn activates the formation of mTOR, phosphorylates S6K1, and inhibits 4EBP1 activity [42]. The phosphorylation of S6K1 promotes the initiation of translation, while 4E-BP1 is a negative regulator of the translation process [43]. *igf-1* is significantly highly expressed in fish liver [44]. Although the expression of *igf-1* and *s6k1* in liver was not significantly affected in this experiment, the difference in *mtor* still indicated that the substitution of CAP for fishmeal would still affect the IGF-1/PI3K/AKT/mTOR signaling pathway to some extent. It has been reported that replacing fishmeal with blended alternatives leads to down-regulation of the expression of *mtor*, *s6k1* in grouper [45], which is similar to the present study. In addition, the expression of these two genes in grouper is also affected by certain amino acid deficiencies [46]. Subject to IGF-1 mediated effects, changes in the expression level of *myhc* result in changes in the content of myosin [44]. In a previous study on pearl gentian grouper, Yang et al. [47] used its expression as an index to evaluate muscle protein synthesis capacity. In this study, the expression level of *mtor*, *4ebp1*, *s6k1*, and *myhc* in dorsal muscles all showed to varying degrees that CAP has a negative effect on muscle protein synthesis at higher levels of pair substitution. After all, CAP has not yet been found to contain active small peptides or unknown growth factors, which are nutrients that are abundant in fishmeal [2].

The height of the intestinal mucosal folds, as well as the length and density of the microvilli determine the absorption surface area of the intestine together [20,48]. In this study, microvilli in the CAP60 group were disorganized, sparse, and short; intestinal folds and muscle layer thickness were also decreased with the increasing CAP levels, similar to the results of studies in *M. salmoides* [16], reflects the damage of the intestinal. In addition, this result of decreased trypsin activity further suggests that the absorption capacity of the intestine would be affected. Based on the results of *akt*, *mtor*, and *s6k1* gene expression assays, it can be further inferred that high levels of CAP substitution have a negative impact on intestinal development. Deficiencies in intestinal digestive and absorptive capacity were important in affecting growth performance in this experiment.

The mucus layer of the intestine has a barrier role in protecting the intestine, and its main component, mucoglycoprotein, is secreted by the goblet cells [49]. Goblet cells are highly differentiated columnar epithelial cells [50] that appear vacuolated after HE staining treatment. In this study, the high number of goblet cells in the CAP15 group versus the control group suggests that there is some improvement in intestinal barrier function by CAP, but further, the high levels of substitution can diminish intestinal barrier function. It has also been reported that exposure to oxidative damage leads to a reduction of intestinal goblet cells in pearl gentian grouper [51]. In this study, the activities of SOD were decreased in the high-level CAP substitution group. All these results suggest that a diet with high levels of CAP substitution for fishmeal also causes some degree of oxidative damage to the distal intestines of pearl gentian grouper. Epinecidin has the ability to kill microorganisms directly or inhibit their growth [52]. In previous studies on grouper, it has been found that the expression level of epinecidin-related genes in the distal intestine can be affected by the vitamin and probiotic preparations added to the feed [53,54]. Combined with the result that the relative expression of *epinecidin* mRNA in distal intestine was upregulated with the increase in feed CAP substitution in this experiment, it is hypothesized that CAP, as a single-cell protein, contains unknown factors that stimulate the distal intestine leading to an increase in epinecidin expression, the mechanism of which needs to be further investigated.

## 5. Conclusions

In this study, CAP replacing 30% of the fishmeal in the diet had no significant negative effect on pearl grouper; when the replacement level was up to 45%, it caused some liver and intestinal health problems but did not significantly affect growth. However, higher levels of substitution (60%) caused a decrease in some non-specific immune indicators in the blood, also caused oxidative damage to the liver and distal intestine, which has a significant negative impact on health, and affected protein synthesis, ultimately reducing the growth of grouper. It was also worth noting that high levels of CAP significantly affected cholesterol and bile acid metabolism in the liver. In conclusion, CAP is a new environmentally friendly protein source suitable for use in pearl gentian grouper feeds. The direction of future research can be new feed formulations by combining CAP with other protein ingredients or adding certain attractants to improve the acceptance of CAP by grouper, in order to increase the application prospects.

## Figures and Tables

**Table 1:** Ingredient composition and nutrient content of experimental diets (dry matter basis).

			Groups		
Ingredients (%)	FM	CAP15	CAP30	CAP45	CAP60
Brown fishmeal	50	42.5	35	27.5	20
CAP	0	6	12	18	24
Gluten	4	4	4	4	4
Soybean meal	13	13	13	13	13
Wheat flour	21	21	21	21	21
Fish oil	2.50	3.16	3.82	4.49	5.15
Soybean oil	2	2	2	2	2
Soybean lecithin	1.5	1.5	1.5	1.5	1.5
Choline chloride	0.5	0.5	0.5	0.5	0.5
Vitamin C	0.1	0.1	0.1	0.1	0.1
CaH_2_PO_4_	1.5	1.5	1.5	1.5	1.5
Compound premix ^1^	1	1	1	1	1
Ethoxyquin	0.05	0.05	0.05	0.05	0.05
Microcrystalline cellulose	2.85	3.57	4.29	5.00	5.73
Methionine	0	0.02	0.03	0.05	0.06
Arginine	0	0.10	0.21	0.31	0.41
Total	100	100	100	100	100
Proximate composition (%) ^2^					
Crude protein	48.45	48.47	48.94	48.44	48.31
Methionine	0.92	0.93	0.95	0.94	0.94
Arginine	2.41	2.41	2.42	2.48	2.45
Crude fat	12.71	12.68	12.61	12.49	11.94
Moisture	9.97	9.48	9.20	9.38	9.10

^1^ Compound premix (g kg^−1^ mixture): vitamin A, 500,000 IU; vitamin D_3_, 100,000 IU; vitamin E, 4.00 g; vitamin K_3_, 1.00 g; vitamin B_1_, 0.50 g, vitamin B_2_, 1.00 g; vitamin B_6_, 1.00 g; vitamin B_12_, 0.002 g; nicotinic acid, 4.00 g; calcium pantothenate, 2.00 g; biotin, 0.01 g; folic acid, 0.10 mg; vitamin C, 15.00 g; ferrum, 10.00 g; cuprum, 0.30 g; zinc, 5.00 g; manganese, 1.20 g; iodine, 0.08 g; cobalt, 0.02 g; selenium, 0.03 g. ^2^ Measured value of dry matter.

**Table 2:** Sequence of the primers used for real-time PCR.

Gene	Forward Primer	Reverse Primer	Genbank NO./ Reference	Fragment Length (bp)
*hmgcr*	AACATGATCTCTAAGGGTAC	CCTCTCTGACCACTTTAG	Xu et al. [23]	187
*fxr*	AGGTGCTTGTGAGTGCCATC	TTCCTCTGCGCTGTACTGTT	Xu et al. [23]	98
*cyp7a1*	GCTACAAGGTGATGTTTG	GTTCTCCAAAGCGTTTAG	Xu et al. [23]	119
*cyp27a1*	ATTGCAGCAGCAAGGAGGTT	TGCGCCAGCTATTGAGACCT	Xu et al. [23]	126
*igf-1*	GTGCGATGTGCTGTATCTCCTG	GCCATAGCCTGTTGGTTTACTG	AY776159.1	179
*akt*	GGCAGGATGTGGTACAGAAGAAGC	TGTCTGGAGGAGTGAGTGTGATGG	Yin et al. [24]	123
*mtor*	TGAGGAGTGGACGCTGGTGAG	CTGCTGAGCCAATGAGAAGAGGTC	Yin et al. [24]	193
*4e-bp1*	GACCACTGCCAAGGCCATC	CTGAACAGGGTTCCTCCGG	JN850959.1	155
*s6k1*	CACCCCTAAAGACTCGCCTG	TTCTTCCCGTGTAGCTGCTG	NM199645.1	159
*keap1*	TTCCATAACGGGGCAACCTC	GGAAGAAGGTCGAGTGCCAA	EF373684.1	128
*nrf2*	CTCGTCAGGCGCTTCTCTAC	GCCGAGATCAATGTCCTGGT	XM018665037.1	115
*epinecidin*	TCACGCTGGCAAGATGATCC	CTCGTTCAAAGGCACGTTGG	KU892416.1	92
*tlr22*	CAAAACTGGAAGGGGAGCAA	GCTCATCAAACAGGCGGAAG	JQ965995.1	144
*il-1β*	CCAGCGTTGAGGGCAGAA	ATCGTCTCCAGATGTAAGGTT	EU219847.1	103
*il-10*	CAGCAGAGTCATTGTCATCTCC	GAGTGGCAATGATCTCAGTCTC	XM033644959.1	117
*tnf-α*	GCTGCGGCTCGAAGACAAT	CAGACGGTGCGGATGGAGT	JN850959.1	219
*myhc*	AAGGTGTTGGCTGAGTGGAAA	TGGATGCTCTTGCCAGTCTCA	NM199645.1	221

*hmgcr,* *3-hydroxy-3-methylglutaryl coenzyme A reductase* *; fxr, farnesoid X receptor; cyp7a1, cholesterol 7α-hydroxylase; cyp27a1, cholesterol 27α-hydroxylase; igf-1, insulin-like growth factor; akt, serine/threonine-protein kinase; mtor, mammalian target of rapamycin; 4e-bp1, eukaryotic translation initiation factor 4E-binding protein 1; s6k1, ribosomal protein S6 kinase 1; nrf2, nuclear erythroid 2-related factor 2; keap1, kelch-like ECH associating protein 1; tlr22, toll-like receptor; tnf-α, tumor necrosis factor α; il-10, interleukin 10; il-1β:* *interleukin 1β* *; myhc, myosin heavy chain.*

**Table 3:** Growth performance of pearl gentian grouper fed gradient substitution of fishmeal in the diet with CAP.

			Groups				Pr(>F) ^1^	
Items	FM	CAP15	CAP30	CAP45	CAP60	A	L	Q
IBW (g)	17.90 ± 0.06	17.87 ± 0.05	17.98 ± 0.03	17.98 ± 0.04	18.10 ± 0.11	0.190	0.037	0.121
FBW (g)	95.20 ± 1.50 ^b^	94.98 ± 0.91 ^b^	92.75 ± 1.81 ^ab^	91.70 ± 0.44 ^ab^	89.60 ± 1.44 ^a^	0.044	0.001	0.006
WG (%)	431.83 ± 8.04 ^b^	431.72 ± 4.13 ^b^	415.87 ± 10.00 ^ab^	410.15 ± 2.62 ^ab^	397.95 ± 8.45 ^a^	0.020	0.001	0.003
SGR (%/d)	2.98 ± 0.03 ^b^	2.98 ± 0.01 ^b^	2.93 ± 0.03 ^ab^	2.91 ± 0.01 ^ab^	2.87 ± 0.03 ^a^	0.022	0.001	0.003
FCR	0.80 ± 0.01	0.80 ± 0.00	0.81 ± 0.01	0.81 ± 0.01	0.81 ± 0.01	0.641	0.627	0.889
DFI (%/d)	1.90 ± 0.01 ^ab^	1.90 ± 0.02 ^ab^	1.91 ± 0.00 ^b^	1.87 ± 0.01 ^ab^	1.86 ± 0.01 ^a^	0.010	0.007	0.005
SR (%)	92.00 ± 1.63	89.25 ± 3.40	95.00 ± 2.52	89.00 ± 1.91	90.00 ± 3.83	0.541	0.636	0.829

IBW: initial body weight; FBW: finial body weight; WG: weight gain; SGR: specific growth rate; FCR: feed conversion ratio; DFI: daily feed intake ratio; SR: survival rate. Mean values within the same row with different superscripts are significantly different (*p* < 0.05). Values = Mean ± pooled standard error of the mean (SEM), *n* = 4. ^1^ Significance probability associated with the F-statistic. A: Analysis of Variance; L: analysis of Linear trend; Q: Analysis of Quadratic trend.

**Table 4:** Whole fish composition of pearl gentian grouper-fed gradient substitution of fishmeal in the diet with CAP.

			Groups				Pr(>F) ^1^	
Items	FM	CAP15	CAP30	CAP45	CAP60	A	L	Q
Body condition indices								
CF (kg/cm^3^ %)	2.71 ± 0.10	2.88 ± 0.03	2.74 ± 0.09	2.86 ± 0.11	2.92 ± 0.02	0.277	0.295	0.585
HSI (%)	3.17 ± 0.10 ^a^	3.18 ± 0.12 ^a^	3.25 ± 0.07 ^a^	3.28 ± 0.11 ^a^	3.83 ± 0.10 ^b^	0.001	0.002	0.007
VSI (%)	9.60 ± 0.22 ^a^	9.91 ± 0.13 ^ab^	10.00 ± 0.33 ^ab^	10.01 ± 0.04 ^ab^	10.67 ± 0.10 ^b^	0.019	0.002	0.008
Body compositions (wet weight)								
Moisture (%)	73.20 ± 0.46	72.48 ± 0.23	72.68 ± 0.08	72.96 ± 0.19	72.53 ± 0.19	0.321	0.347	0.543
Crude protein (%)	16.58 ± 0.29	16.94 ± 0.16	16.74 ± 0.28	16.69 ± 0.15	16.91 ± 0.18	0.757	0.538	0.822
Crude fat (%)	5.32 ± 0.08	5.24 ± 0.16	5.43 ± 0.22	5.29 ± 0.20	5.45 ± 0.11	0.866	0.530	0.813

CF: condition factor; HSI: hepatosomatic index; VSI: viscerosomatic index. Mean values within the same row with different superscripts are significantly different (*p* < 0.05). Values = Mean ± pooled standard error of the mean (SEM), *n* = 4. ^1^ Significance probability associated with the F-statistic. A: Analysis of Variance; L: analysis of Linear trend; Q: Analysis of Quadratic trend.

**Table 5:** Serum biochemical indices and enzyme activities of pearl gentian grouper-fed gradient substitution of fishmeal in the diet with CAP.

			Groups				Pr(>F) ^1^	
Items	FM	CAP15	CAP30	CAP45	CAP60	A	L	Q
TP (g/L)	56.89 ± 2.38	59.33 ± 3.45	58.19 ± 2.71	60.36 ± 1.80	58.74 ± 2.63	0.912	0.552	0.740
TG (mmol/L)	0.56 ± 0.02	0.53 ± 0.02	0.54 ± 0.06	0.54 ± 0.03	0.58 ± 0.05	0.829	0.517	0.512
T-CHO (mmol/L)	2.24 ± 0.11^c^	1.73 ± 0.05 ^ab^	2.16 ± 0.11 ^bc^	1.39 ± 0.16 ^a^	1.28 ± 0.10 ^a^	0.000	0.000	0.000
UA (μmmol/L)	42.95 ± 2.47	44.90 ± 4.19	37.09 ± 1.95	39.04 ± 2.90	40.02 ± 3.18	0.320	0.230	0.390
MDA (nmol/L)	5.97 ± 0.71 ^a^	7.73 ± 0.29 ^ab^	9.39 ± 0.31 ^bc^	10.71 ± 0.34 ^c^	10.26 ± 0.65 ^c^	0.000	0.000	0.000
GSH (μmmol/L)	13.03 ± 0.66 ^ab^	13.63 ± 0.48 ^b^	12.13 ± 0.87 ^ab^	11.16 ± 0.97 ^ab^	9.96 ± 0.49 ^a^	0.031	0.002	0.009
T-AOC (mM)	0.27 ± 0.01	0.25 ± 0.01	0.26 ± 0.02	0.27 ± 0.02	0.28 ± 0.01	0.631	0.252	0.158
AST (U/mL)	14.44 ± 1.10 ^ab^	16.86 ± 2.93 ^ab^	17.50 ± 2.07^ab^	21.46 ± 0.64 ^b^	12.45 ± 1.60 ^a^	0.023	0.928	0.043
ALT (U/mL)	73.24 ± 2.51 ^a^	72.38 ± 1.48 ^a^	82.07 ± 1.88 ^b^	82.4 ± 2.31 ^b^	85.34 ± 1.87 ^b^	0.000	0.000	0.000
ACP (U/L)	18.42 ± 1.23 ^a^	17.62 ± 1.13 ^a^	20.44 ± 1.34 ^a^	23.35 ± 1.78 ^ab^	26.88 ± 1.67 ^b^	0.001	0.000	0.000

TP: total protein; TG: triglyceride; T-CHO: total cholesterol; UA: uric acid; MDA: malondialdehyde; GSH: glutathione; T-AOC: total antioxidant capacity; AST; aspartate aminotransferase; ALT: alanine aminotransferase; ACP: acid phosphatase. Mean values within the same row with different superscripts are significantly different (*p* < 0.05). Values = Mean ± pooled standard error of the mean (SEM), *n* = 4. ^1^ Significance probability associated with the F-statistic. A: Analysis of Variance; L: analysis of Linear trend; Q: Analysis of Quadratic trend.

**Table 6:** Biochemical indices and enzyme activities in liver and distal intestines of pearl gentian grouper-fed gradient substitution of fishmeal in the diet with CAP.

			Groups				Pr(>F) ^1^	
Items	FM	CAP15	CAP30	CAP45	CAP60	A	L	Q
Liver								
MDA (nmol/mgprot)	6.32 ± 0.07 ^a^	5.74 ± 0.49 ^a^	7.73 ± 0.85 ^ab^	9.18 ± 0.73 ^ab^	11.29 ± 1.23 ^b^	0.003	0.000	0.000
GSH (μmol/gprot)	25.02 ± 1.27 ^b^	25.37 ± 1.74 ^b^	23.37 ± 1.70 ^ab^	18.88 ± 0.92 ^ab^	18.85 ± 1.08^a^	0.014	0.001	0.004
T-AOC (mmol/gprot)	1.15 ± 0.09 ^b^	1.00 ± 0.03 ^ab^	0.92 ± 0.02 ^ab^	0.91 ± 0.08 ^ab^	0.83 ± 0.08 ^a^	0.047	0.002	0.007
ACP (U/gprot)	84.31 ± 3.18 ^a^	78.63 ± 4.16 ^a^	96.46 ± 1.21 ^ab^	113.07 ± 9.58 ^b^	101.13 ± 5.63 ^ab^	0.009	0.007	0.027
SOD (U/mgprot)	9.90 ± 0.38	8.63 ± 0.07	9.59 ± 0.46	8.66 ± 0.40	8.10 ± 0.84	0.135	0.046	0.144
Lysozyme (U/gprot)	37.33 ± 3.74	36.66 ± 1.07	32.86 ± 2.52	31.86 ± 2.07	32.81 ± 1.64	0.141	0.018	0.043
Distal Intestine								
Trypsin (U/mgprot)	418.82 ± 10.29^b^	404.03 ± 10.4^ab^	380.60 ± 9.48^a^	386.53 ± 5.03^a^	378.93 ± 9.02^a^	0.046	0.005	0.009
SOD U/mgprot	5.91 ± 0.03^b^	5.69 ± 0.06^ab^	5.54 ± 0.09^a^	5.46 ± 0.09^a^	5.49 ± 0.03^a^	0.004	0.000	0.000
Lysozyme U/gprot	31.96 ± 0.93	30.49 ± 0.55	32.37 ± 1.08	30.93 ± 0.37	30.05 ± 0.21	0.186	0.189	0.337

MDA: malondialdehyde; GSH: glutathione; T-AOC: total antioxidant capacity; ACP: acid phosphatase; SOD: superoxide dismutase. Mean values within the same row with different superscripts are significantly different (*p* < 0.05). Values = Mean ± pooled standard error of the mean (SEM), *n* = 4. ^1^ Significance probability associated with the F-statistic. A: Analysis of Variance; L: analysis of Linear trend; Q: Analysis of Quadratic trend.

**Table 7:** Distal intestinal morphological histology of pearl gentian grouper-fed gradient substitution of fishmeal in the diet with CAP (HE-stained sections).

			Groups				Pr(>F) ^1^	
Items	FM	CAP15	CAP30	CAP45	CAP60	A	L	Q
mucosal fold height (μm)	530.18 ± 7.97 ^b^	518.80 ± 7.40 ^ab^	512.93 ± 9.18 ^ab^	499.54 ± 8.21 ^a^	491.58 ± 7.66 ^a^	0.008	0.000	0.001
mucosal fold width (μm)	83.75 ± 2.36	83.54 ± 2.04	80.80 ± 2.10	82.43 ± 2.17	80.42 ± 2.09	0.735	0.254	0.521
muscularis thickness (μm)	100.40 ± 3.19 ^ab^	103.40 ± 1.42 ^b^	96.68 ± 2.47 ^ab^	94.08 ± 2.61 ^ab^	92.83 ± 2.66 ^a^	0.026	0.001	0.001
goblet cell relative number^2^	16.33 ± 0.67 ^b^	17.23 ± 0.57 ^b^	15.37 ± 0.82 ^ab^	15.47 ± 0.80 ^ab^	13.33 ± 0.90 ^a^	0.008	0.001	0.002

Values = Mean ± pooled standard error of the mean (SEM), *n* = 4. ^1^ Significance probability associated with the F-statistic. A: Analysis of Variance; L: analysis of Linear trend; Q: Analysis of Quadratic trend. ^2^ Goblet cell relative number is the number of goblet cells per 200 nm mucosal fold. Mean values within the same row with different superscripts are significantly different (*p* < 0.05).

**Table 8:** Distal intestinal morphological histology of pearl gentian grouper fed gradient substitution of fishmeal in the diet with CAP (TEM).

			Groups				Pr(>F) ^1^	
Items	FM	CAP15	CAP30	CAP45	CAP60	A	L	Q
microvillus height (μm)	2.19 ± 0.03 ^b^		2.09 ± 0.04 ^ab^		2.00 ± 0.04 ^a^	0.001	0.000	0.001
microvillus density (count/μm)	6.26 ± 0.08 ^b^		6.19 ± 0.06 ^b^		5.84 ± 0.10 ^a^	0.003	0.002	0.003

Values = Mean ± pooled standard error of the mean (SEM), *n* = 4. ^1^ Significance probability associated with the F-statistic. A: Analysis of Variance; L: analysis of Linear trend; Q: Analysis of Quadratic trend. Mean values within the same row with different superscripts are significantly different (*p* < 0.05).

## Data Availability

The data that support the findings of this study are available on request from the corresponding author. The data are not publicly available due to privacy or ethical.

## Supplementary Materials

The following supporting information can be downloaded at: https://www.mdpi.com/article/10.3390/ani13061090/s1, Table S1: Nutrition composition of CAP and brown fishmeal (air-dried basis).
