# Supplementary material for: Effects of Fishmeal Replacement by Clostridium Autoethanogenum Protein Meal on Cholesterol Bile Acid Metabolism, Antioxidant Capacity, Hepatic and Intestinal Health of Pearl Gentian Grouper (Epinephelus Fuscoguttatus ♀ × Epinephelus Lanceolatus ♂)
Source: Animals (Basel). 2023 Mar 18;13(6):1090. doi: 10.3390/ani13061090 (PMC10044235; doi:10.3390/ani13061090)
Supplement: Supplementary file 1 [file animals-13-01090-s001.zip › animals-2202876-supplementary.pdf]

Supplementary Material

Effects of fishmeal replacement by *Clostridium autoethanogenum* protein meal on cholesterol bile acid metabolism, antioxidant capacity, hepatic and intestinal health of pearl gentian grouper (*Epinephelus fuscoguttatus* ♀ × *Epinephelus lanceolatus* ♂)

Bocheng Huang[1], Menglin Shi[1], Aobo Pang[1], Beiping Tan[1,2,3], Shiwei Xie[1,2,3,4*]

*** Correspondence:** Shiwei Xie xswzsdx@163.com

Table S1 Nutrition composition of CAP and brown fishmeal (air-dried basis).

Items	Brown fish meal	CAP	Unit	Note
Moisture	6.8	7.14	%	
Calcium	3.01	0.125	%	
Ferrum	2400	970	mg/kg	
Magnesium	2900	240	mg/kg	
Manganese	11	0	mg/kg	
Potassium	10000	3000	mg/kg	
Sodium	16000	810	mg/kg	
Zinc	78	72	mg/kg	
Selenium	1.72	38.18	mg/kg	
Cobalt	0.241	7.03	mg/kg	
Iodine	0.357	0.411	mg/kg	
Total phosphorus	2.31	0.99	%	
Crude protein	68.21	84.14	%	
Methionine	2.98	2.72	%	content in CP
Cystine	0.60	0.84	%	content in CP
Lysine	7.64	10.33	%	content in CP
Arginine	5.99	4.04	%	content in CP
Threonine	4.21	4.77	%	content in CP
Asparagine	8.95	11.33	%	content in CP
Hydroxyalanine	3.83	3.81	%	content in CP
Glutamic acid	12.83	11.61	%	content in CP

Glycine	6.06	4.60	%	content in CP
Alanine	6.48	5.50	%	content in CP
Valine	4.93	6.46	%	content in CP
Isoleucine	4.02	6.27	%	content in CP
Leucine	7.71	7.58	%	content in CP
Tyrosine	3.36	3.73	%	content in CP
Phenylalanine	5.27	3.92	%	content in CP
Histidine	3.04	2.00	%	content in CP
Proline	4.16	2.85	%	content in CP
Crude fat	9.00	0.19	%	

Abbreviations: CAP: *Clostridium autoethanogenum* protein meal; CP: crude protein.
